# Antifungal synergy of a topical triazole, PC945, with a systemic triazole against respiratory *Aspergillus fumigatus* infection

**DOI:** 10.1038/s41598-019-45890-w

**Published:** 2019-07-01

**Authors:** Thomas Colley, Gurpreet Sehra, Leah Daly, Genki Kimura, Takahiro Nakaoki, Yuki Nishimoto, Yasuo Kizawa, Pete Strong, Garth Rapeport, Kazuhiro Ito

**Affiliations:** 1 Pulmocide Ltd, London, SW7 2PG UK; 2 Laboratory of Physiology and Anatomy, Nihon University School of Pharmacy, Funabashi, Chiba 274-8555 Japan

**Keywords:** Drug delivery, Antifungal agents

## Abstract

Invasive pulmonary Aspergillosis is a leading cause of morbidity and mortality in immunosuppressed patients and treatment outcomes using oral antifungal triazoles remain suboptimal. Here we show that combining topical treatment using PC945, a novel inhaled triazole, with systemic treatment using known triazoles demonstrated synergistic antifungal effects against *Aspergillus fumigatus* (AF) in an *in vitro* human alveolus bilayer model and in the lungs of neutropenic immunocompromised mice. Combination treatment with apical PC945 and either basolateral posaconazole or voriconazole resulted in a synergistic interaction with potency improved over either compound as a monotherapy against both azole-susceptible and resistant AF invasion *in vitro*. Surprisingly there was little, or no synergistic interaction observed when apical and basolateral posaconazole or voriconazole were combined. In addition, repeated prophylactic treatment with PC945, but not posaconazole or voriconazole, showed superior effects to single prophylactic dose, suggesting tissue retention and/or accumulation of PC945. Furthermore, in mice infected with AF intranasally, 83% of animals treated with a combination of intranasal PC945 and oral posaconazole survived until day 7, while little protective effects were observed by either compound alone. Thus, the combination of a highly optimised topical triazole with oral triazoles potentially induces synergistic effects against AF infection.

## Introduction

Inhalation and deposition of *Aspergillus* conidia in the lung is the most common route of infection for patients who contract invasive aspergillosis^1^. The recommended treatment for invasive aspergillosis is given orally or intravenously^2^, and the outcomes are still suboptimal. Considering the site of infection, exposure of the original infection site to high levels of antifungals should have benefits as orally treated triazoles do not achieve sustained high concentrations in the lung lumen. It has been shown that aerosolised delivery of antifungals to the lung results in higher concentrations of compound in the epithelial lining fluid and bronchial alveolar lavage, when compared to intravenous delivery^3^. In recent years several studies have shown potential inhalation therapy with known antifungals^4–6^, but as those compounds were not optimised for inhalation therapy, they were absorbed systemically quickly rather than having an extended residence time lung^7^. PC945 is a novel antifungal triazole, designed specifically for inhaled administration with sustained lung residency and persistent antifungal activity^8^, and will be a powerful tool to investigate the concept of combination therapy with topical and systemic azoles.

To evaluate the effects of antifungal combinations, standard susceptibility testing in microtiter plates is often used. However, this system does not differentiate topical and systemic treatment. Also, it is not translational as human host cells are not present. Recently, *in vitro* models of the human alveolus have been developed to understand the kinetics underlying *A. fumigatus* infection and penetration from the lung into the systemic compartment^9^. The system consists of two chambers divided by a porous membrane separating an apical layer of alveolar epithelial cells from a basolateral layer of vascular endothelial cells. This system allows a topical compound to be administered apically in upper chamber and/or an oral compound basolaterally in lower chamber. In this report we describe the synergistic effects of combined treatment with topical (PC945) and systemic triazoles against penetration of azole-susceptible and resistant *A. fumigatus*. In addition, the effects of repeated prophylactic treatment of PC945 was investigated and compared with those of posaconazole and voriconazole to discriminate PC945 from known triazoles based on longer cell residency as well as accumulation, and also as this is a key component for the prophylactic treatment of immunocompromised patients.

## Results

### Anti-fungal activities of PC945 and comparators on *A. fumigatus* strains by broth microdilution microplate assay

PC945 exhibited the most potent antifungal activity on azole-susceptible strain NCPF2010 with the MIC value of 0.063 µg/mL but did not show MIC for TR34/L98H and TR46/Y121F/T289A strains (MIC: >16 µg/mL) by standard EUCAST MIC assay (Supplement Table S1). However, quantitative analysis where the fungus growth was determined by OD, revealed more than 50% inhibition of fungal growth by PC945 in those azole-resistant strains (Supplement Table S2). NCPF2010 was also susceptible to posaconazole, voriconazole and itraconazole, but those compounds showed no or little effects on TR34/L98H and TR46/Y121F/T289A strains (Supplement Tables S1 and S2).

### *In vitro* antifungal activity of apically treated triazoles against *A. fumigatus* in the human alveolus model

*A. fumigatus* conidia were added to the upper chamber (epithelial compartment) and galactomannan (GM) concentrations in the lower chamber (endothelial compartment) were measured to monitor penetration of the bilayer by fungus. Non-infective control did not show any GM, suggesting no contamination during experiments even though the inserts were located in same plate that contained the inserts for infection control (Supplement Fig. S1). In addition, DMSO (0.5% in final) did not affect the level of GM in lower chamber as the marker of fungus invasion (Supplement Fig. S1).

**Figure 1:**
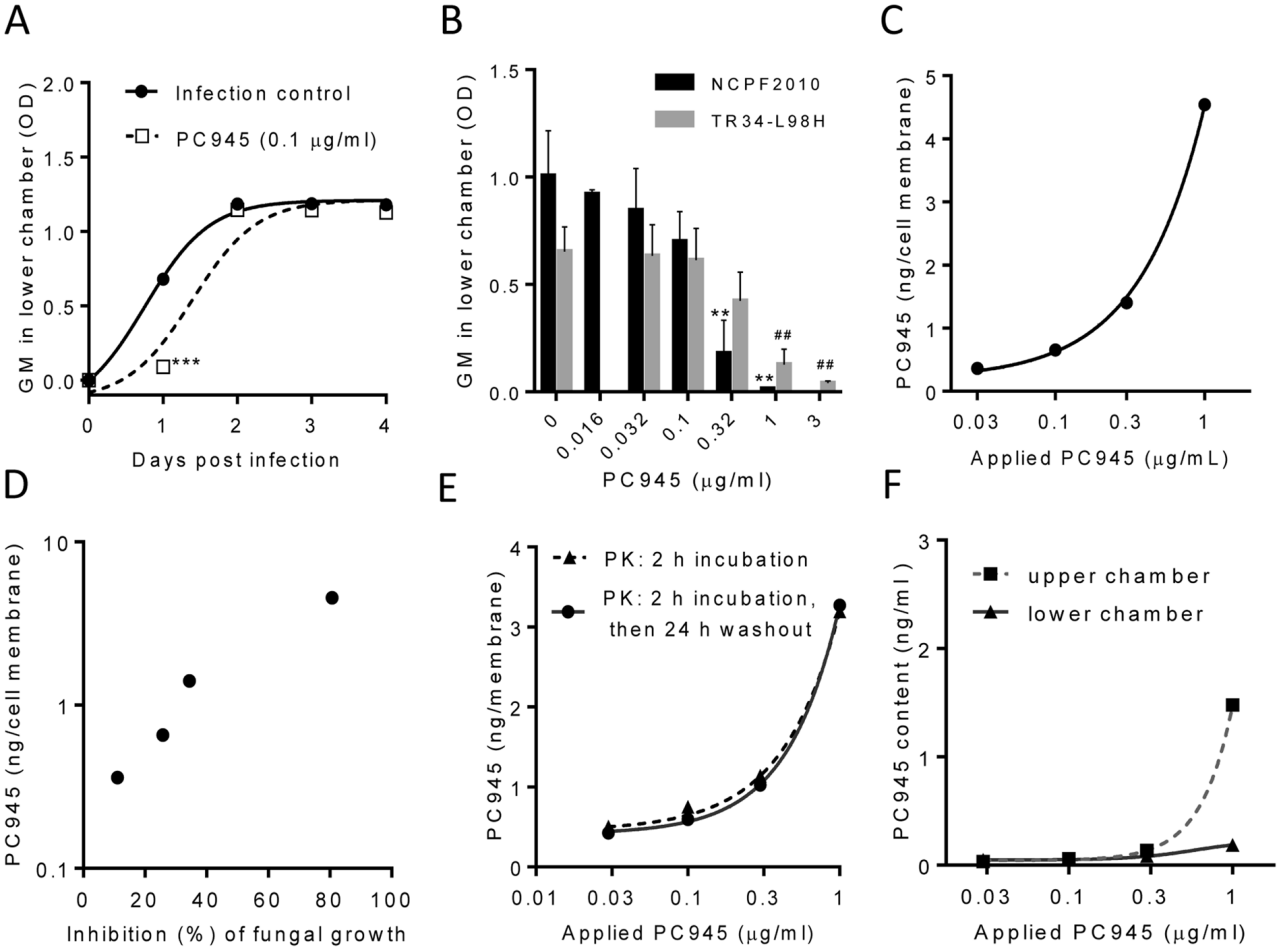
Pharmacodynamics and pharmacokinetics of PC945 in an *in vitro* model of the human alveolus. (**A**) Time-dependent change of GM in lower chamber after azole-susceptible *A. fumigatus* infection to the upper chamber, n = 3, ***p < 0.001 vs. control (**B**) Concentration-dependent change of GM in lower chamber 24 h after azole-sensitive *A. fumigatus* (NCPF2010; ■) or azole-resistant *A. fumigatus* (TR_34_/L98H; □) inoculation to the upper chamber, n = 3, **p < 0.01 vs NCPF2010 control, ^##^p < 0.01 vs. L98H control (**C**) PC945 in cell membrane recovered 24 h after treatment to the upper chamber. (**D**) Correlation of PC945 cellular content with inhibition of *A. fumigatus* penetration to the lower chamber, (**E**) Retention of PC945 in the bilayer when treated for 2 h (▲) or 2 h plus 24 h washout (●), (**F**) PC945 in media of upper (■) and lower (▲) chamber recovered 24 h after treatment to the upper chamber.

As shown in Fig. 1A, the level of GM in the lower chamber significantly increased 24 h post inoculation of azole-susceptible *A. fumigatus* strain (NCPF2010) and increased further up to 72 h (3 days) post inoculation. PC945 (0.1 μg/ml), when administered to the upper chamber to mirror inhalation treatment of the lung, inhibited the levels of GM, particularly during first 24 h (Fig. 1A).

The PC945’s inhibition seen 24 h post inoculation of *A. fumigatus* (NCPF2010 strain) was concentration-dependent (Fig. 1B), and it was observed that PC945 (IC_50_ = 0.17 μg/ml, IC_90_ = 0.41 μg/ml) (Fig. 1B) was 3.6 and 2.2-fold more potent than voriconazole (0.61 μg/ml, 0.91 μg/ml), respectively, and comparable to or less potent than posaconazole (0.11 μg/ml, 0.15 μg/ml) and itraconazole (0.074 μg/ml, 0.40 μg/ml), when administered to the upper chamber, at inhibiting azole-susceptible *A. fumigatus* penetration of the bilayer (Supplementary Fig. S2, Supplement Table S3).

In addition, PC945 concentration-dependently inhibited the penetration of azole-resistant TR_34_/L98H *A. fumigatus* through the bilayer (IC_50_ = 0.41 μg/ml, IC_90_ = 1.45 μg/ml) (Fig. 1B) when treated in upper chamber. PC945 was 3.4 and >2.1-fold more potent than voriconazole (1.38 μg/ml, >3 μg/ml, respectively) and comparable to itraconazole (0.37 μg/ml, 2.3 μg/ml, respectively), while posaconazole (0.16 μg/ml, 0.40 μg/ml) was also an effective inhibitor (Supplement Fig. S2, Supplement Table S3).

For optimization of combination experiments, we also evaluated the effects of lower chamber treatment of posaconazole, voriconazole and itraconazole. All compounds showed more potent effects than upper chamber treatment and those compounds also showed beneficial effects even in TR34/L98H infection (Supplementary Fig. S3, Supplement Table S3).

### Pharmacokinetic profile of PC945

After a 2 h incubation with PC945, administered to the upper chamber, cell bilayers were removed after washout and PC945 content in the cell bilayer construct was analysed. As observed in Fig. 1C, PC945 content was higher in cells treated with higher concentrations, and the recovery rate (cell content/total compounds applied) was 3.2%, 3.8%, 7.5% and 12% at 1, 0.3, 0.1, 0.03 μg/ml, respectively. More importantly, the measured concentrations correlated well with the inhibitory effects of *A. fumigatus* penetration (NCPF2010) in the same experiment (Fig. 1D).

Subsequently the cell bilayer construct was collected 26 h after treatment (2 h compound treatment followed by a 24 h washout period). As shown in Fig. 1E, washout was found to have no effect on PC945 cellular concentrations, suggesting sustained residency of PC945 in cells. We also confirmed that only limited PC945 was detected in the lower chamber and PC945 was more abundant in the upper chamber (Fig. 1F). Therefore, the site of action of PC945 on *A. fumigatus* penetration was the apical epithelial rather than the endothelial compartment.

### Combined treatment and assessment of synergy against azole-susceptible *A. fumigatus* in the human alveolus model

**Figure 2:**
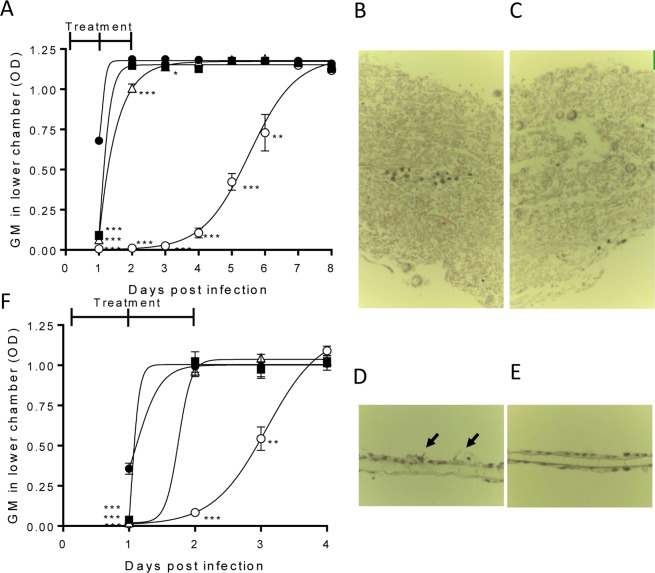
Combined PC945 and posaconazole treatment within an *in vitro* model of the human alveolus. (**A**) Time course comparison of compounds alone or combined against azole-susceptible *A. fumigatus* (NCPF2010) penetration of the bilayer, vehicle infection control (●), PC945 (0.1 μg/mL, upper, ■), posaconazole (0.01 μg/ml, lower, Δ), combination (○). n = 3 (**B**–**E**) Representative images of histological analysis of *A. fumigatus* (NCPF2010) infected bilayer, with mono- or combined compound administration, [haematoxylin & eosin staining (x400)], (**B**) *A. fumigatus* infection control, (**C**) posaconazole (0.01 μg/ml, lower), (**D**) PC945 (0.1 μg/ml, upper) and (**E**) combined. Arrows: fugal body, (**F**) Time course comparison of compounds alone or in combination against azole-resistant *A. fumigatus* (TR_34_/L98H) penetration of the bilayer, vehicle infection control (●), PC945 (1 μg/mL, upper, ■), posaconazole (0.1 μg/ml, lower, Δ), combined (○). n = 3 *p < 0.05, **p < 0.01, ***p < 0.001 vs. control.

PC945 or other triazoles were administered to the upper chamber to mimic topical inhaled treatment, while either posaconazole or voriconazole was administered to the lower chamber to mimic systemic exposure after oral treatment. PC945 treatment (0.1 µg/ml) of the upper chamber showed inhibition of *A. fumigatus* penetration by 86.4 ± 4.47% on Day 1, dropping to 3.36 ± 1.26% on Day 2 (Fig. 2A). Posaconazole (0.01 µg/ml) treated in the lower chamber showed 90.5 ± 2.79% inhibition on Day 1, dropping to 15.0 ± 0.96% on Day 2 (Fig. 2A). In contrast, combined treatment with PC945 (upper chamber) and posaconazole (lower chamber) achieved >50% inhibition of bilayer *A. fumigatus* penetration up until Day 5 (Table 1, Fig. 2A), with a calculated synergy ratio (SR) of >1, demonstrating a sustained synergistic effect. The synergistic effect of combined PC945 (upper chamber) and posaconazole (lower chamber) was confirmed by histology (Fig. 2B–E). In infection control, *Aspergillus* overgrew and destroyed the cell bilayer (Fig. 2B) on Day 3 post inoculation. Posaconazole (0.01 µg/ml) did not inhibit growth of *Aspergillus* (Fig. 2C), and PC945 (upper chamber) partially inhibited it as some fungus growth were observed (Fig. 2D, arrow). In contrast, combined treatment with PC945 (upper chamber) and posaconazole (lower chamber) strongly inhibited fungal growth and no change of host cell morphology was observed (Fig. 2E). These beneficial effects were also observed with the combinations of PC945-itraconazole and PC945-voriconazole (Supplementary Fig. S5).

**Table 1:** Effects of combination treatment on inhibition of *A. fumigatus* (NCPF2010) invasion.

Compartment Treated		Days (% Inhibition)							
Upper	Lower	1	2	3	4	5	6	7	8
PC945 (0.1 μg/mL)	DMSO	86.4 ± 4.47^d^	3.36 ± 1.26	3.65 ± 0.38	4.47 ± 1.27	0.30 ± 0.59	−0.31 ± 2.28	0.90 ± 1.65	2.76 ± 1.43
DMSO	Posaconazole (0.01 μg/mL)	90.5 ± 2.79^d^	15.0 ± 0.96^d^	4.14 ± 0.82^b^	1.10 ± 0.63	−0.55 ± 1.86	−1.13 ± 2.49	0.25 ± 0.47	0.82 ± 0.28
PC945 (0.1 μg/mL)	Posaconazole (0.01 μg/mL)	98.9 ± 0.03^d^	99.1 ± 0.06^d^	97.8 ± 0.81^d^	91.0 ± 2.56^d^	64.1 ± 4.79^c^	38.2 ± 8.50	2.13 ± 1.5	3.73 ± 1.29
	SR^a^	1.00 ± 0.00	**5.57** ± **0.33**	**13.0** ± **1.16**	**20.6** ± **7.70**	**136** ± **85.6**	**97.2** ± **87.4**	**5.61** ± **5.06**	1.61 ± 0.63
Posaconazole (0.1 μg/mL)	DMSO	88.4 ± 2.89^d^	9.59 ± 3.78^b^	1.13 ± 0.51	−0.26 ± 1.41	−1.16 ± 1.33	0.34 ± 1.50	−0.06 ± 0.57	1.85 ± 1.70
DMSO	Posaconazole (0.1 μg/mL)	95.8 ± 0.61^d^	99.1 ± 0.03^d^	99.2 ± 0.00^d^	99.1 ± 0.07^d^	97.5 ± 1.29^d^	83.4 ± 9.94^d^	−4.24 ± 1.25	17.2 ± 2.38
Posaconazole (0.1 μg/mL)	Posaconazole (0.1 μg/mL)	95.6 ± 0.69^d^	99.2 ± 0.00^d^	99.2 ± 0.00^d^	99.2 ± 0.00^d^	99.0 ± 0.12^d^	97.1 ± 0.84^d^	9.04 ± 8.24	12.3 ± 2.96
	SR^a^	0.96 ± 0.01	1.00 ± 0.00	1.00 ± 0.00	1.00 ± 0.00	1.02 ± 0.01	1.20 ± 0.16	16.4 ± 7.87	0.64 ± 0.05
Voriconazole (1 μg/mL)	DMSO	95.0 ± 0.23^d^	88.8 ± 6.04^d^	59.4 ± 24.7^b^	30.8 ± 31.0	−1.61 ± 1.04	−1.67 ± 2.31	−0.55 ± 1.73	3.81 ± 1.71
DMSO	Voriconazole (1 μg/mL)	95.9 ± 0.37^4^	99.1 ± 0.03^d^	99.2 ± 0.07^d^	94.8 ± 2.19^d^	−1.82 ± 1.01	−0.67 ± 2.92	6.92 ± 5.06	4.37 ± 3.31
Voriconazole (1 μg/mL)	Voriconazole (1 μg/mL)	95.5 ± 0.00^d^	99.1 ± 0.03^d^	99.3 ± 0.07^d^	94.9 ± 2.13^d^	−3.13 ± 0.20	−1.93 ± 3.84	7.74 ± 4.98	3.33 ± 2.16
	SR^a^	0.96 ± 0.00	0.99 ± 0.00	1.00 ± 0.00	0.98 ± 0.05	0.42 ± 0.08	0.63 ± 0.13	1.34 ± 0.59	0.41 ± 0.07

N = 3 of independent experiments, each experiment was conducted in duplicate.

^a^Synergic Ratio (>1: Synergy), The value of 0.1 was applied for 0 or negative values to assist in the calculations.

^b^p < 0.05, ^c^p < 0.01, ^d^p < 0.001 vs. infection control.

In contrast, when posaconazole was administered to the upper and lower chamber in combination, only mild synergistic effects were seen on Day 6 and 7 (Table 1). Similarly, when voriconazole was administered to both compartments in combination, no synergistic effect was seen until Day 5 (Table 1). Furthermore, whilst synergism was indicated based on the SR value on Day 7, the level of inhibition was low (SR = 1.34 ± 0.59; 7.74 ± 0.59% inhibition). In addition, when both posaconazole and PC945 were applied to only the upper chamber or both posaconazole and PC945 were applied to only the lower chamber, no synergic effects were observed (Supplement Fig. S6A,B). When PC945 was treated in both upper chamber and lower chamber in combination, additive/synergic effects were observed (Supplement Fig. S6C), suggesting that synergy were observed only when PC945 was treated in upper chamber.

To evaluate molecular based interaction, a checker board analysis of antifungal activities of PC945 and posaconazole/voriconazole/itraconazole were conducted in broth microdilution microplate assay, and it demonstrated no marked synergic effects although additive or minor synergic effects were observed at random (Supplement Table S4), suggesting no direct interaction on targeting molecules.

### Combined treatment and assessment of synergy against azole-resistant *A. fumigatus* in the human alveolus model

We also investigated the benefit of combined treatment against azole-resistant *A. fumigatus* harbouring TR_46_/Y121F/T289A and *A. fumigatus* harbouring TR_34_/L98H. PC945 at 3 µg/ml only partially inhibited the penetration of *A. fumigatus* (TR_46_/Y121F/T289A) on Day 1 by 29.7 ± 2.05% and showed no effects from Day 2 (Table 2). Posaconazole (0.3 µg/ml lower chamber) showed marked inhibition on Day 1 and Day 2 only. However, combined treatment with PC945 (upper chamber) and posaconazole (lower chamber) resulted in a greater inhibition of *A. fumigatus* bilayer penetration, up to Day 4 post inoculation (SR = 43.2 ± 33.4; 88.9 ± 4.67% inhibition) (Table 2), whilst voriconazole-voriconazole combination did not show any synergistic effects. The effects of the high concentration of posaconazole (3 µg/ml, lower chamber) were too marked to allow potential synergistic effects to be evaluated.

**Table 2:** Effects of combination treatment on inhibition of multi-azole-resistant TR_46_/Y121F/T289A *A. fumigatus* invasion.

Compartment Treated		Days (% Inhibition)					
Upper	Lower	1	2	3	4	5	6
PC945 (3 μg/mL)	DMSO	29.7 ± 2.05^c^	−0.16 ± 1.97	−3.12 ± 3.78	4.22 ± 1.78	−12.5 ± 2.99	4.64 ± 4.42
DMSO	Posaconazole (0.3 μg/mL)	98.1 ± 0.03^c^	81.4 ± 1.83^c^	26.9 ± 15.7	1.94 ± 1.16	−10.7 ± 4.73	5.94 ± 2.91
PC945 (3 μg/mL)	Posaconazole (0.3 μg/mL)	98.9 ± 0.20^c^	96.8 ± 0.44^c^	93.8 ± 1.04^b^	88.9 ± 4.67^c^	−7.38 ± 5.02	2.78 ± 5.05
	SR^a^	1.00 ± 0.00	1.19 ± 0.02	**6.61** ± **3.45**	**43.2** ± **33.4**	0.50 ± 0.00	1.00 ± 0.00
Posaconazole (3 μg/mL)	DMSO	99.1 ± 0.26^c^	97.2 ± 1.33^c^	93.6 ± 3.72^c^	52.1 ± 15.6^b^	−6.69 ± 5.22	5.78 ± 2.24
DMSO	Posaconazole (3 μg/mL)	99.8 ± 0.12^c^	99.9 ± 0.03^c^	99.9 ± 0.00^c^	99.9 ± 0.03^c^	99.8 ± 0.09^c^	7.32 ± 1.96
Posaconazole (3 μg/mL)	Posaconazole (3 μg/mL)	99.7 ± 0.13^c^	99.9 ± 0.03^c^	99.9 ± 0.03^c^	99.9 ± 0.03^c^	99.7 ± 0.03^c^	0.15 ± 1.60
	SR^a^	1.00 ± 0.00	1.00 ± 0.00	1.00 ± 0.00	1.00 ± 0.00	1.00 ± 0.00	0.07 ± 0.06
Voriconazole (10 μg/mL)	DMSO	−4.71 ± 0.90	1.57 ± 4.76	−0.99 ± 4.58	2.56 ± 2.10	−4.04 ± 0.33	1.78 ± 3.99
DMSO	Voriconazole (10 μg/mL)	16.1 ± 12.5	−1.32 ± 1.61	−1.23 ± 3.92	−4.06 ± 4.75	−6.01 ± 5.10	6.45 ± 3.96
Voriconazole (10 μg/mL)	Voriconazole (10 μg/mL)	23.1 ± 6.42	4.25 ± 2.80	−5.86 ± 2.90	−3.97 ± 2.07	−12.0 ± 1.58	5.31 ± 3.19
	SR^a^	**17.9** ± **16.8**	**8.75** ± **8.28**	0.18 ± 0.16	0.04 ± 0.01	0.41 ± 0.09	0.58 ± 0.19

N = 3 independent experiments, each experiment was conducted in duplicate.

^a^Synergic Ratio (>1: Synergy), The value of 0.1 was applied for 0 or negative values to assist in the calculations.

^b^p < 0.01, ^c^p < 0.001 vs. infection control.

On *A. fumigatus* harbouring TR_34_/L98H, PC945 (1 µg/ml, upper chamber) and posaconazole (0.1 µg/ml, lower chamber) achieved 88.8 ± 1.24% and 94.2 ± 0.66% inhibition of TR_34_/L98H *A. fumigatus* bilayer penetration at Day 1, respectively, but were much reduced in effectiveness by Day 2 (Fig. 2F). In contrast, combined treatment with PC945 and posaconazole achieved >50% inhibition of bilayer penetration up until day 2 (91.6 ± 2.44% inhibition). The effects were also observed in the combination of PC945 and voriconazole, and PC945 and itraconazole (Supplement Fig. S7), but synergy was not observed in a combination of posaconazole/posaconazole or voriconazole/voriconazole (Supplementary Table S5).

### *In vivo* potency of combined therapy on mice infected with azole susceptible *A. fumigatus*

**Figure 3:**
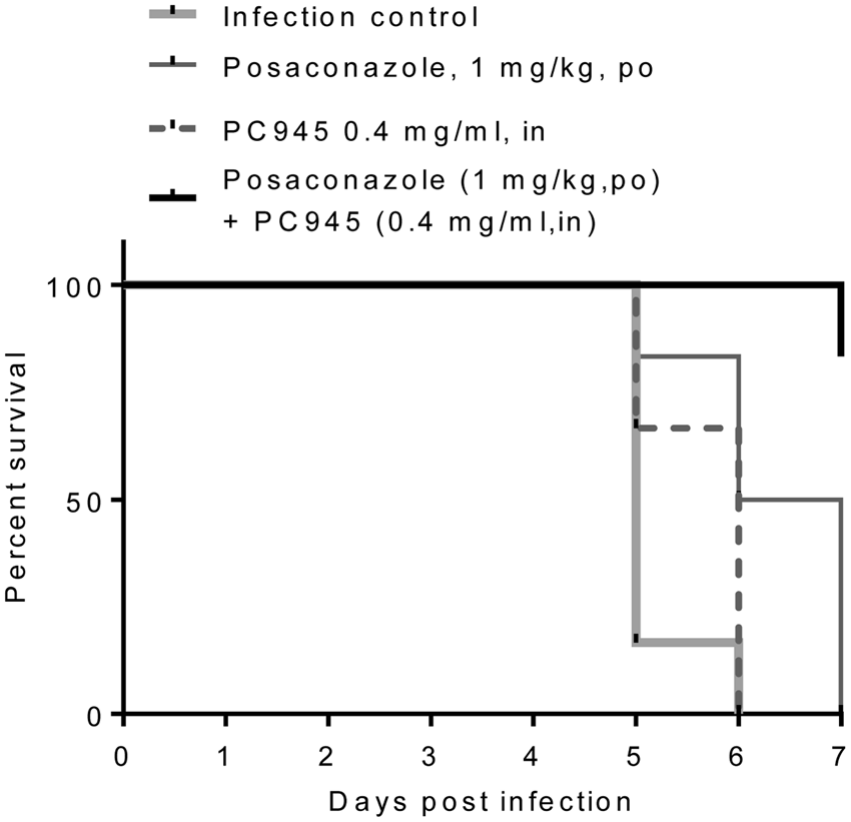
Effects of once-daily intranasal treatment with PC945 (0.4 mg/ml, in) and posaconazole (1 mg/kg, po) or combined treatment (0.4 mg/ml PC945 in +1 mg/kg posaconazole po) on survival of *A. fumigatus*-infected immunocompromised mice (n = 6 each group).

PC945 was administered intranasally and posaconazole orally to immunocompromised neutropenic mice daily from one day after azole-susceptible *A. fumigatus* (ATCC-13073 strain) intranasal inoculation. The suboptimal doses were selected based on several *in vivo* experiments previously reported^8,10^. No control mice survived beyond day 7, with the median survival being 5 days (Fig. 3, Supplement Table S6). Similarly, no mice dosed intranasally with 0.4 mg/ml PC945 (approximately, 0.56 mg/kg, in) or orally with 1 mg/kg posaconazole survived to Day 7, with the median survival time being 6 and 6.5 days, respectively. Strikingly, mice treated with combined intranasal PC945 and oral posaconazole exhibited a significantly higher survival rate at day 7 (83%; median survival: undefined, p < 0.001 vs infection control, p < 0.01 vs. posaconazole alone or PC945 alone, Table S6-1). These *in vivo* effects were confirmed in another experiment (Table S6-2).

### Prophylactic antifungal effects against an azole-susceptible strain of *A. fumigatus*

**Figure 4:**
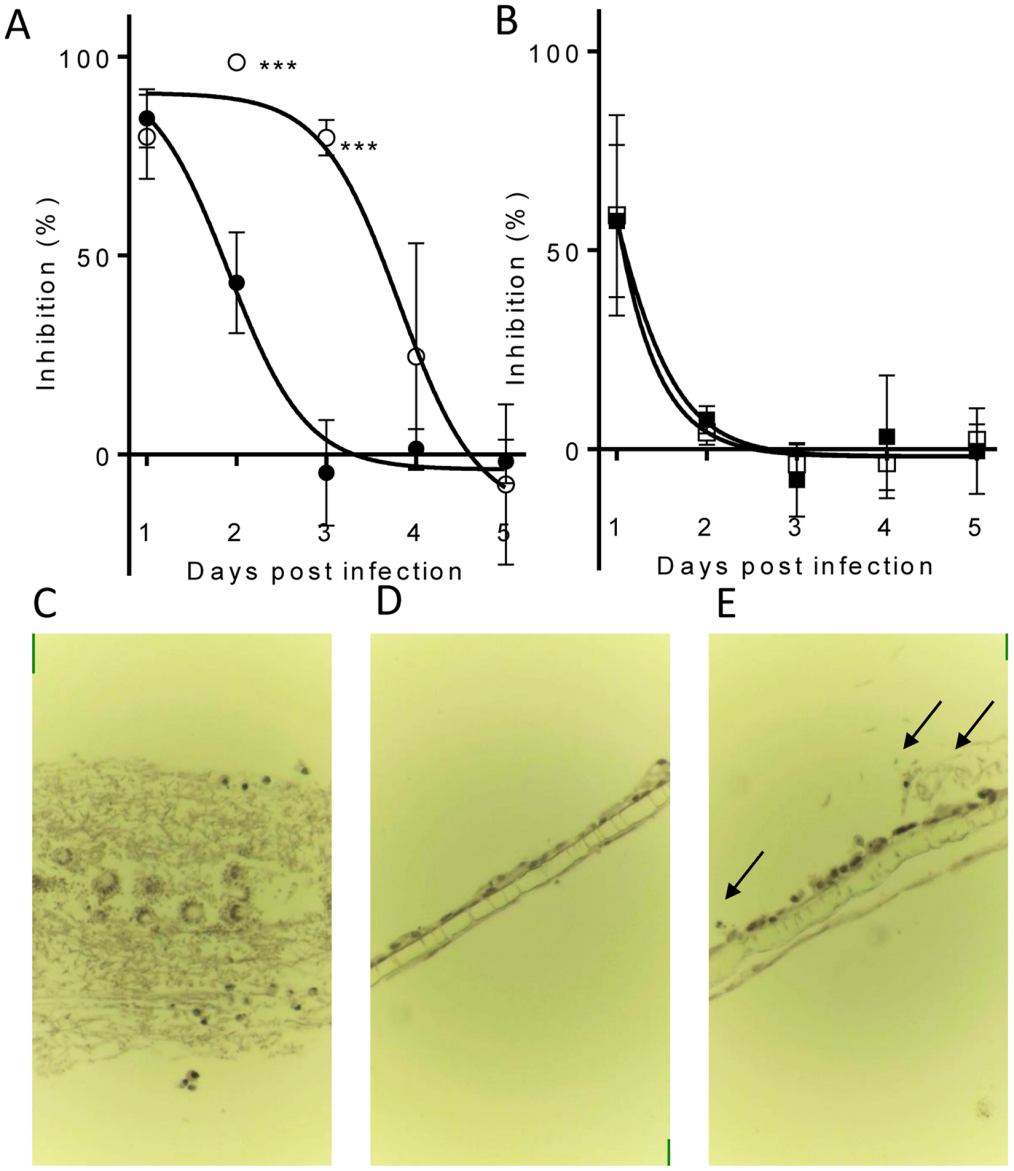
Prophylactic treatment inhibition of *A. fumigatus* (NCPF2010) penetration to the human alveolus. (**A**) PC945 (0.3 μg/ml, upper) 24 h prophylaxis (●) versus 4 days (−96, −72, −48, −24 h) repeated prophylaxis (○). n = 3 **p < 0.01, ***p < 0.001 vs. 24 h prophylaxis, (**B**) posaconazole (0.3 μg/ml, upper) 24 h prophylaxis (●) versus 4 days repeated prophylaxis (○), n = 3, (**C**–**E**) Representative images of histological analysis [haematoxylin & eosin staining (x400)], (**C**) *A. fumigatus* infection control, (**D**) PC945 (0.3 μg/ml, upper x4 days), (**E**) posaconazole (0.3 μg/ml, upper, x4 days). Arrow: fungal body.

Inhaled therapy with an appropriate molecule is useful as a prophylactic treatment, since limited adverse effects will occur due to limited systemic exposure. The efficacy of prophylactic administration of compounds was investigated using the *in vitro* model of the human alveolus described above. Administration of compounds daily for 4 days (repeat treatment regimen at 96, 72, 48, 24 h prior to infection) prior to infection with azole-susceptible *A. fumigatus* (NCPF2010) was compared against a single treatment (single treatment regimen) given 24 h prior to infection. To quantify the impact of each treatment regimen, GM concentrations in the lower chamber were measured daily for 5 days after infection with *A. fumigatus*, and the day at which fungal burden reached 50% of the control (DFB_50_) was calculated from the inhibition curves. Single treatment with PC945 achieved 84.4 ± 4.22% inhibition on Day 1 after inoculation, and the inhibitory activity reduced over time (DFB_50_ = 1.85 ± 0.16). Repeated prophylactic treatment with PC945 significantly increased antifungal activity versus single treatment (DFB_50_ = 3.58 ± 0.27; p < 0.01) (Table 3, Fig. 4A). In contrast, repeated prophylaxis with either posaconazole (0.3 µg/mL) or voriconazole (10 µg/mL) did not show improved activity (Table 3, Fig. 4B). Additional experiments demonstrated that posaconazole did not enhance anti-fungal activity by 7 days repeated dosing with higher concentrations (1 µg/mL) (Supplement Table S7). The superior prophylactic effect of PC945 versus posaconazole was confirmed by histology (Fig. 4C–E). Under control conditions, *Aspergillus* overgrew and destroyed the cell bilayer (Fig. 4C). Prophylaxis with posaconazole partially inhibited the growth of *Aspergillus* (Fig. 4E), but PC945 completely inhibited it (Fig. 4D).

**Table 3:** Prophylactic antifungal effects of PC945 and known antifungal agents against NCPF2010, an azole-susceptible strain of *A. fumigatus*.

Test Agent	Concentration (μg/mL)	Treatment regime	Days (Galactomannan inhibition [%])					DFB_50_
			1	2	3	4	5	
PC945	0.3	Single	84.4 ± 4.22	43.1 ± 7.33	−4.57 ± 7.69	1.44 ± 2.87	−1.78 ± 3.17	1.85 ± 0.16
		Repeat	79.8 ± 6.11	98.5 ± 0.43^a^	79.5 ± 2.58^b^	24.5 ± 16.4	−7.57 ± 11.6	3.58 ± 0.27^b^
Posaconazole	0.3	Single	58.7 ± 14.5	4.20 ± 1.81	−3.74 ± 2.86	−3.51 ± 3.93	2.34 ± 2.22	<1.53
		Repeat	57.3 ± 11.0	7.43 ± 2.00	−7.72 ± 5.33	3.13 ± 8.95	−0.51 ± 6.20	<1.31
Voriconazole	10	Single	89.5 ± 2.97	34.3 ± 23.6	−0.34 ± 2.93			1.79 ± 0.27
		Repeat	86.4 ± 5.03	−1.41 ± 3.12	0.58 ± 2.05			1.09 ± 0.06

DFB_50_ = Days until fungal burden reaches 50% of control. Single: treated at 24 h prior to fungus inoculation, Repeat: treated at 96, 72, 48, 24 h prior to fungus inoculation.

^a^p < 0.05, ^b^P < 0.01; PC945 single treatment vs repeat treatment.

## Discussion

Although combination treatment with antifungal compounds with different modes of action has been well documented by fungal culture in microplates, we differentiated for the first time the synergistic antifungal effects of different treatment routes for antifungal triazoles.

The *in vitro A. fumigatus* infection model of the human alveolus has been well characterised by Hope *et al*. and other groups^9,11–14^. This system offers several advantages for experimental design that can be exploited while assessing the antifungal activity of novel agents or combination strategies. Firstly, the characteristic apical facing surface of bilayer cultures allows for direct application of topical agents, such as PC945, to the surface of infected cells, so serving as a model for the planned route of delivery *in vivo*. Oral agents can also be added to the lower chamber (endothelial compartment) to mimic systemic exposure. Secondly, by measuring GM in the lower chamber, the level of invasion/penetration through the bilayer can be assessed. The model reflects the reality of *A. fumigatus* infection of the lung in immunocompromised patients, where colonisation of the alveolar epithelium is followed by penetration and angioinvasion, resulting in invasive disease, compared to broth microdilution using microplates without host cells.

PC945 is the first triazole antifungal to be developed specifically for inhaled or topical delivery, allowing direct administration to the site of infection with limited systemic exposure^10^. Known triazoles have been repurposed for inhalation therapy, but are absorbed systemically quickly after inhalation^7^. In contrast, PC945 showed persistent biological action in epithelial cells and in the hyphal fungal body^8^, which we also confirmed by observing anti-fungal activity and cellular pharmacokinetics in the current study (Fig. 1A,E). As shown in Fig. 1F, PC945 persisted in cells and was not absorbed significantly into the lower chamber in this model. Furthermore, PC945’s biological effects accumulated after repeated dosing (current study and previous publication^10^), a desirable feature for prophylactic treatment. Interestingly, we did not find any accumulation of biological effects by posaconazole or voriconazole, suggesting that both compounds would be less attractive candidates for prophylactic inhaled delivery. Thus, PC945 is well optimised for topical therapy, and the best tool to investigate the combined treatment with topical and systemic triazoles using *in vitro* and *in vivo* models.

A major finding in the current study is the antifungal synergy seen when PC945 was topically treated and posaconazole was added to the lower compartment. Furthermore, synergy was not specific to this combination, since topical PC945 combined with either itraconazole or voriconazole in the endothelial compartment, showed similar effects. However, very interestingly, the synergy was not observed in either the upper posaconazole-lower posaconazole or upper voriconazole-lower voriconazole combinations (Supplement Tables 1, 2 and S5). Combining nebulized amphotericin B with oral fluconazole or injectable amphotericin B was reported to result in favourable clinical outcomes in lung transplant recipients^15,16^. Other studies have shown that combined systemic azole-echinocandin, AMB-echinocandin or other combined antifungal treatment regimens can improve survival when compared to monotherapy^15–18^. Whilst these studies used a combination of antifungals with different modes of action, the current study investigated the potential of combined topical triazole and systemic triazole having same molecular target for the treatment of pulmonary Aspergillosis. In fact, in broth microdilution microplate assay, a checker board analysis did not prove strong synergy of PC945 and posaconazole/voriconazole/itraconazole (Supplement Table S4). In the bilayer system, when both posaconazole and PC945 were applied to upper chamber only or when both posaconazole and PC945 were applied to lower chamber only, it did not show synergic effects (Supplement Figs. S6A and S6B). In addition, when PC945 was applied to both upper and lower chamber in combination, moderate to strong synergy was observed (Supplement Fig. S6C). Since, we do not expect meaningful systemic exposure of PC945 after inhaled dosing and PC945 is not orally bioavailable, we do not think this finding in Fig. S6C is relevant to the clinical setting. Thus, synergy was observed only when PC945 was used for apical treatment, and it could not be explained by interaction of target molecules. Topical treatment could eradicate colonized fungi in the airways although not once the fungus invaded systemically. Systemically treated anti-fungal effectively eliminates fungi although the limitation of systemic triazoles in the treatment of an airway’s infection is well documented elsewhere. Thus, topical and systemic azoles can support each other to combat serious *Aspergillus* infection. In addition, use of a topical triazole potentially has the benefit of lowering the required dose of oral triazoles, minimising the unwanted side effects of oral triazole treatment. Thus, this approach should be safer than using high dose oral triazole as a monotherapy, as well as offering enhanced antifungal activity.

This antifungal synergy achieved by combination of PC945 with a systemic triazole was confirmed *in vivo* in *A. fumigatus* infected, completely neutropenic immunocompromised mice, where either topical PC945 or oral posaconazole showed limited protection. We previously reported that intranasal PC945 was effective against *A. fumigatus* infection in “temporarily” neutropenic mice and discussed the possibility that the antifungal effects of PC945 are augmented by immune cells^8,10^. The effects of PC945 monotherapy were limited in the completely neutropenic invasive aspergillosis model shown in Fig. 3, due to the aggressive systemic invasive infection and no protection by immune cells. Even oral posaconazole required a higher dose to improve survival in this model compared with that used in temporarily neutropenic mice (Supplement Table S6)^19^. However, in this completely neutropenic immunocompromised mouse model, combination treatment significantly improved the survival of mice infected with *A. fumigatus*. This data is reproducible (Supplement Table S6) and consistent with that observed using the *in vitro* human alveolus model, which like severely immunocompromised patients, has no immune cells in the system. These data support a combined treatment approach, where PC945 is delivered locally to the lung and posaconazole is delivered orally in immunocompromised patients.

The widespread use of azole antifungal agents, both in the clinic and in agriculture, has led to a growing and problematic emergence of resistant mycoses^20–22^. *A. fumigatus* isolates with environmentally acquired resistance mechanisms, including TR_34_/L98H and TR_46_/Y121F/T289A, were first discovered in the Netherlands in 1998 but are now recognised to be a world-wide health concern^23^. Recently it has been reported that 3.2% of clinical *A. fumigatus* isolates are azole-resistant^24^, however, in some European countries, resistance rates are even higher. For instance, a multicentre survey from the Netherlands reported itraconazole resistance in up to 6.0% of clinical *A. fumigatus* isolates^21^. Therefore, identifying effective therapeutic regimens for resistant mycoses is a growing priority^25,26^. Based on the results shown (Fig. 2F, Table 2, Supplement Table S5, Supplement Fig. S7), combined therapy with topical and systemic azoles is a potential treatment for pulmonary aspergillosis caused by azole-resistant pulmonary *A. fumigatus*.

Azole antifungal prophylaxis is often used in neutropenic patients with haematological malignancies as this patient group is at an increased risk of mortality if treatment is delayed^27^. The current study demonstrated that repeat-dosing prophylaxis with posaconazole or voriconazole had no added beneficial inhibitory effect on fungal burden when compared against a single treatment. However, repeated treatment with PC945, at an identical concentration to posaconazole, extended the inhibitory effect from 1.85 ± 0.16 to 3.58 ± 0.27 days. We demonstrated PC945 to be well retained within bronchial epithelial cells in the human alveolus model (Fig. 4, Table 3, Supplement Table S7), while extended prophylaxis enhanced the antifungal activity of PC945 in temporarily neutropenic mice infected with *A. fumigatus*, providing evidence for compound accumulation within lung cells^8,10^.

There are several limitations of our current study. Firstly, fungal growth and antifungal effects of PC945 have not been monitored time-dependently in bilayer system histologically. Therefore, we don’t know how fungi penetrates the bilayer and whether PC945 can inhibit fungal penetration beyond the epithelial cell layer. Consequently, it is difficult to compare with clinical set up. Further time-dependent histology study is required. Secondly, in the *in vivo* study, we treated compounds only a day after fungus inoculation, and additional study to evaluate the effects of delayed therapeutic treatment, which likely happens in clinic, will be required. Thirdly, due to less timepoint of sample collection, it is inconclusive whether PK-PD relationship is linear or sigmoidal. Further *in vitro* PK study is required, and also PK-PD should be evaluated in future proof of concept clinical study.

Thus, these data suggest that the combination of topical PC945 triazole with known systemic oral triazoles is a more effective treatment option for immunocompromised patients infected with *A. fumigatus*, and inhaled PC945 has the potential to be an effective prophylactic therapy. This is also potentially safer option as use of a topical triazole potentially has the benefit of lowering the required dose of oral triazoles, minimising the unwanted side effects of oral triazole treatment. Interestingly, the first case report describing the successful use of PC945 to treat a refractory Aspergillus bronchial anastomotic infection and tracheobronchitis in a lung transplant on top of standard care has recently been published^28^. PC945 is now in Phase 2 clinical development in man^29^, and further clinical studies with relevant patient populations are planned.

## Methods

Antifungal agents PC945 was synthesised by Sygnature Discovery Ltd (Nottingham, UK), and voriconazole (Tokyo Chemical Industry UK Ltd., Oxford, UK), itraconazole (Arkopharma, Carros, France), posaconazole (Apichem Chemical Technology Co., Ltd., Zhejiang, China) and amphotericin B (Selleckchem, Munich, Germany) were procured from commercial sources. For *in vitro* antifungal assays, stock solutions of test agents were prepared in DMSO (2000 μg/ml). For *in vivo* studies, solid materials of test agents were directly suspended in physiological saline at 10 mg/ml and diluted with physiological saline after sonication for intranasal treatment.

Broth Microdilution Minimum inhibitory concentration (MIC) values against *Aspergillus fumigatus* NCPF2010, TR_34_/L98H, TR_46_/Y121F/T289 strains, using the 96-well formatted EUCAST E.DEP 9.3. method.^30,31^ Plates were incubated for 48 h at 35 °C, and MIC was read visually, or turbidity was assessed by measuring optical density (OD) at 530 nm using a spectrophotometer. The IC_50_ and IC_90_ values were calculated from the concentration-response curve generated for each test compound. A checker board analysis was also conducted using same protocol.

Cell culture Human pulmonary endothelial cells (HPAEC; Lonza, Slough, UK) were cultured at 37 °C, 5% CO_2_ in endothelial medium (EBM-2; Lonza, Slough, UK) supplemented with EGM-2 SingleQuots. Human alveolar epithelial cell line (A549) were cultured at 37 °C, 5% CO_2_ in Dulbecco’s Modified Eagle’s medium (DMEM) supplemented with 10% FBS and L-glutamine.

*In vitro* model of the human alveolus An *in vitro* model of the human alveolus was constructed, as described^9^, to assess the impact of compounds delivered via the inhaled route and the systemic route. Briefly, HPAEC were seeded (100 μl/well) at a density of 1 × 10^6^ cells/ml onto the under surface of the membrane of transwells (6.5 mm-diameter, 3.0 μm pores; Corning, NY, USA) and incubated at room temperature, within a flow hood, for 2 h. Transwells were righted and set into 24-well plates containing EGM-2 (600 μl), and EGM-2 (100 μl) was added to the upper chamber. After 48 h incubation at 37 °C, 5% CO_2_, A549 cells were seeded (100 μl) into the upper chamber at 0.5 × 10^6^ cells/ml, in EBM-2 supplemented with 10% FBS. All experiments were performed on day 5 after addition of A549 cells.

### *In vitro* antifungal activity against *A. fumigatus* using a model of the human alveolus

The culture media was replaced with 0% FBS EBM-2 in the A549 chamber, and with 2% EBM-2 in the HPAEC chamber before each experiment. Subsequently test agent or vehicle (DMSO) was administered to the upper chamber and the plates were incubated for 1 h at 37 °C, 5% CO_2_. *A. fumigatus* conidia were added to the upper chamber across the plate at a final concentration of 1 × 10^4^ spores/ml and plates were incubated for 24 h at 35 °C, 5% CO_2_. Fungal invasion was determined by collecting supernatants from the lower chamber and measuring GM concentrations, using Platelia GM-EIA kits (Bio-Rad Laboratories, Hemel Hempstead, UK).

Compartment dependent effects of test agent, and combination studies Transwells were set-up as described and the culture media was replaced with 0% FBS EBM-2 in the A549 chamber, and with 2% FBS EBM-2 in the HPAEC chamber. Subsequently test agent or vehicle (DMSO, 0.5% in final) in media was administered to the upper or lower chambers and the plates were incubated for 1 h at 37 °C, 5% CO_2_. *A. fumigatus* conidia were then added to the upper chamber across the plate at a final concentration of 1 × 10^4^ spores/ml and plates were incubated for 24 h at 35 °C, 5% CO_2_. On Day1, after the supernatants in lower chamber were collected, the media were replaced with freshly prepared compound solution. This was repeated on Day 2. Supernatants from the lower chamber were collected daily further after Day2, and the media was refreshed. GM concentrations were assessed using Platelia GM-EIA kits.

Prophylactic antifungal effects of test agents For single prophylaxis, the media in the upper chamber was aspirated and media containing the test articles or DMSO was added (100 µL in 0% FBS EBM-2/well). Transwells were then transferred into a 24-well plate containing 2% FBS EBM-2 (600 µL/well) and the plates were incubated at 37 °C, 5% CO_2_ for 24 h. On day of infection, media was changed and *A. fumigatus* (NCPF2010) conidia suspension was then added to the upper chamber across the plate at a final concentration of 1 × 10^4^ spores/ml. Supernatant from the lower chamber was collected daily and stored at −80 °C for the GM assay using Platelia GM-EIA kits, and the media was refreshed daily.

For repeated prophylaxis, compounds in media were administered to upper chamber at 96, 72, 48, 24 h prior to infection. At each time, the media were replaced with freshly prepared test agents. The percentage inhibition for each well on each day was calculated and the DFB_50_ (days until fungal burden reached 50% of the control) was calculated.

Histology of the human alveolus model Bilayer inserts were collected on Day 2 post conidia inoculation. After gentle apical washing with PBS, the inserts were fixed with 4% paraformaldehyde (upper and lower compartments) at 4 °C for 24 h, and then stored in PBS at 4 °C until use. Fixed bilayer with membrane was removed from inserts and embedded in paraffin wax, which was sectioned with a microtome at 5 microns. The prepared paraffin sections were stained with haematoxylin & eosin (Gills III haematoxylin, Leica Biosystems, Milton Keynes, UK; 0.5% Eosin, Pioneer Research Chemical, Colchester, UK), and observed using an optical microscope (Trinocular MAGNUM-T, #MIC0107, Scientific Laboratory Supplies Limited, Nottingham, UK).

Assay of PC945 content in cells PC945 was administered to the upper epithelial chamber and incubated for 2 h at 37 °C, 5% CO_2_. For the non-washout model, the transwell membrane was carefully separated from the transwell scaffold, transferred to a 1.5 ml tube and snap-frozen on dry ice. For the washout model, the upper and lower chambers were aspirated and washed once with PBS, after which fresh media was added. Following 24 h incubation at 37 °C, 5% CO_2_, the transwell membrane was isolated as described above. Assays to determine the concentration of PC945 were conducted by LGC Limited (Fordham, Middlesex, UK). Briefly, frozen cells with membrane were sonicated in 300 µL of methanol for 5 mins, and centrifuged. The supernatant (250 µL) was removed and blown dry. The sample was reconstituted in 150 µL of MeCN:water (50:50, v/v) and the level of PC945 measured by LC-MS/MS (LLQ was 10 pgmL^−1^, ULQ was 20,000 pgmL^−1^).

*In vivo* antifungal activity against *A. fumigatus* infection. Specific pathogen-free A/J mice (male, 5 weeks old) were purchased from Sankyo Labs Service Co. Ltd. (Tokyo, Japan) and adapted for 1 week in a temperature (24 ± 1 °C) and humidity (55 ± 5%) controlled room, under a 12 h day-night cycle. The mice were reared on a standard diet and tap water *ad libitum*. Animals were then dosed with hydrocortisone (Sigma H4881, 125 mg/kg, subcutaneously) on days −3, −2 and −1 before infection, and with cyclophosphamide (Sigma C0768; 250 mg/kg, intraperitoneally) two days before infection and one day post-infection to induce neutropenia. To avoid bacterial infection, drinking water was supplemented with tetracycline hydrochloride (Sigma T7660; 1 μg/ml) and ciprofloxacin (Fluka 17850; 64 μg/ml).

*A. fumigatus* (ATCC 13073 [strain: NIH 5233], the American Type Culture Collection, Manassas, VA, USA) was grown on malt agar (Nissui Pharmaceutical, Tokyo, Japan) plates for 6–7 days at room temperature (24 ± 1 °C). Conidia were aseptically dislodged from the agar plates and suspended in sterile distilled water with 0.05% Tween 80 and 0.1% agar. On the day of infection, conidial counts were assessed by haemocytometer and the inoculum was adjusted to obtain a concentration of 1.67 × 10^8^/mL in physiological saline. On day 0, 30 μl of the conidia suspension was administered intranasally. Posaconazole, suspended in 20% polyethylene glycol 400 (PEG400) in isotonic saline at a concentration of 0.2 mg/mL (1 mg/5 ml/kg), was administered orally on days 1 to 6. PC945, suspended in physiological saline at a concentration of 0.4 mg/mL, was administered intranasally (35 μl) on days 1 to 6. The survival of animals was recorded for 7 days. Deaths and the body weights of surviving animals were monitored daily. A body weight loss of 20% or more, compared with an animal’s weight on a day before, or a mouse death, were both defined as “drop-out” events. Animals that lost ≥20% of their body weight were sacrificed. All animal studies were approved by the Ethics Review Committee for Animal Experimentation of Nihon University, and all experiments were performed in accordance with relevant guidelines and regulations. *A. fumigatus* studies were approved by the Microbial Safety Management Committee of Nihon University School of Pharmacy (E-H25-001).

### Statistical analysis

For *in vitro* assays, results are expressed as means ± standard error of the mean (SEM). For comparison between groups the ordinary one-way ANOVA with Tukey’s *post hoc* comparison was used. Statistical significance was defined as *P* < 0.05.

The percentage inhibition for each well was calculated and the IC_50_ and IC_90_ values were calculated from the concentration-response curve generated for each test compound.

Synergistic interactions were determined using the Abbott formula^32^, where synergistic interactions are present if the ratio between the experimentally observed efficacy (%*C*_obs_) and the expected efficacy (%*C*_exp_) is greater than 1 [%*C*_exp_ = *A* + *B* − (*AB*/100)]. The value of 0.1 was applied for 0 or negative values to assist in the calculations.

*In vivo* survival analysis was performed by Kaplan-Meier plots followed by the log rank (Mantel-Cox) tests using the PRISM 6^®^ software program (GraphPad Software Inc., San Diego, CA, USA). Statistical significance was defined as *P* < 0.05.

## Supplementary information

Supplement Figures

Supplement Tables
