# Supplementary material for: Antifungal synergy of a topical triazole, PC945, with a systemic triazole against respiratory Aspergillus fumigatus infection
Source: Sci Rep. 2019 Jul 1;9:9482. doi: 10.1038/s41598-019-45890-w (PMC6603190; doi:10.1038/s41598-019-45890-w)
Supplement: Supplementary file 1 — Supplement Figures [file 41598_2019_45890_MOESM1_ESM.pdf]

Supplementary Figures

Title

Antifungal synergy of a topical triazole, PC945, with a systemic triazole against respiratory *Aspergillus fumigatus* infection

Thomas Colley[1], Gurpreet Sehra[1], Leah Daly[1], Genki Kimura[2], Takahiro Nakaoki[2], Yuki Nishimoto[2], Yasuo Kizawa[2], Pete Strong[1], Garth Rapeport[1], Kazuhiro Ito[1*]

[1] Pulmocide Ltd, London, SW7 2PG, UK

[2] Laboratory of Physiology and Anatomy, Nihon University School of Pharmacy, Funabashi, Chiba 274-8555, Japan

* Corresponding author: Dr. Kazuhiro Ito; kaz@pulmocide.com.

Figure S1.

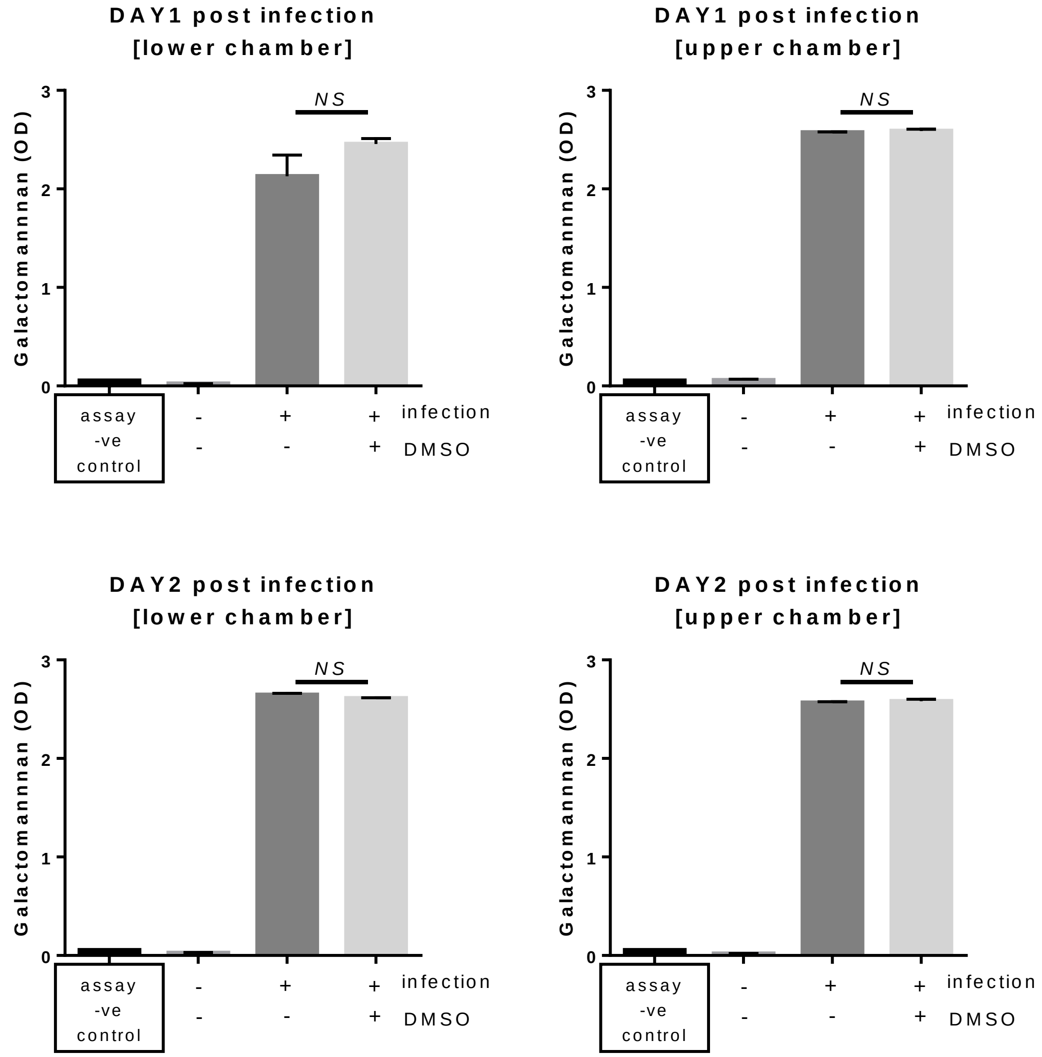

Galactomannan levels in non-infected and infected with/without DMSO on Days 1 and 2 post *A.fumigatus (NCPF2010)* inoculation . Galactomannan was determined in samples collected from upper and lower chambers. (n=3)

Figure S2.

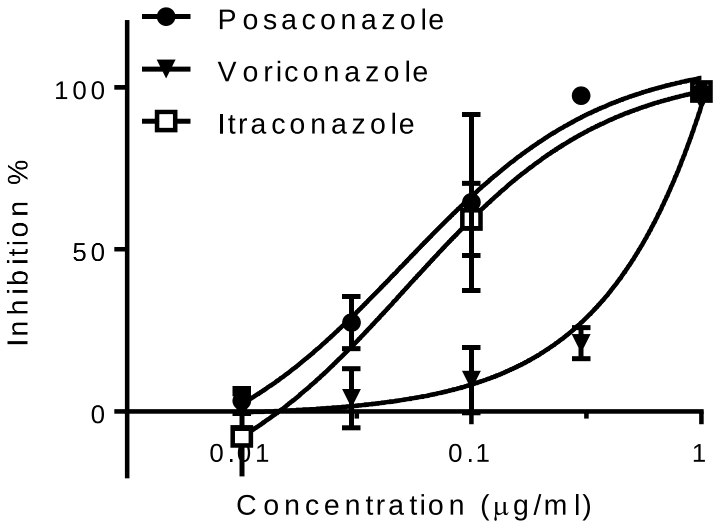

Concentration-dependent inhibition of posaconazole (●), voriconazole (▲) and itraconazole (□) treated in upper chamber on azole-susceptible *A. fumigatus* (NCPF2010) penetration to alveolus model. Galactomannan in the lower chamber was measured a marker of fungus invasion. (n=3)

Figure S3.

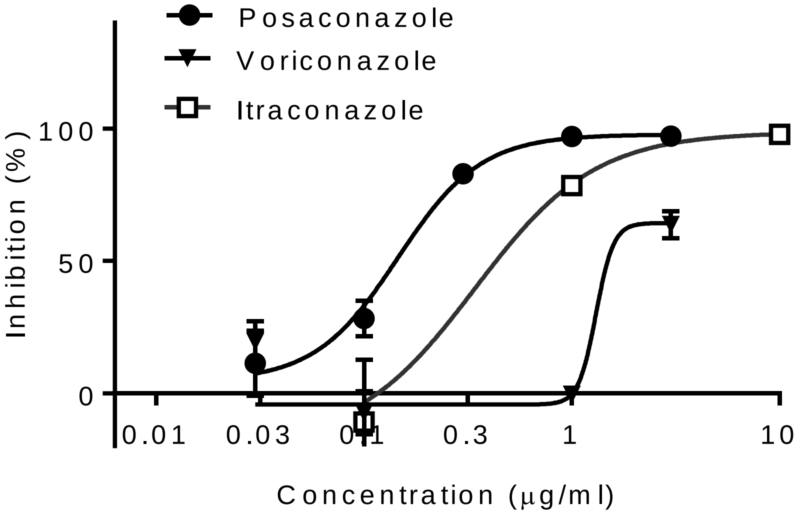

Concentration-dependent inhibition of posaconazole (●), voriconazole (▲) and itraconazole (□) treated in upper chamber on *A. fumigatus* (TR34-L98H) penetration to alveolus model. Galactomannan in the lower chamber was measured a marker of fungus invasion. (n=3)

Figure S4

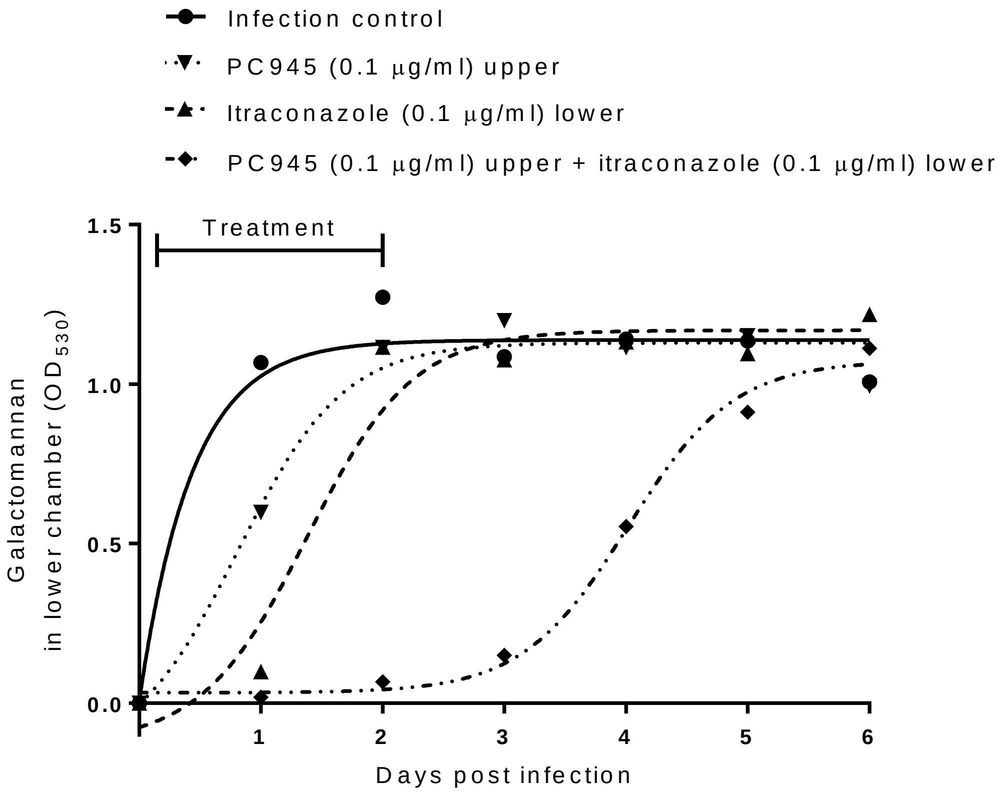

Combined PC945 and itraconazole treatment within an *in vitro* model of the human alveolus. Time course comparison of compounds alone or in combination against azole-sensitive *A. fumigatus (NCPF2010)* penetration of the bilayer. (average of 2 inserts)

Figure S5

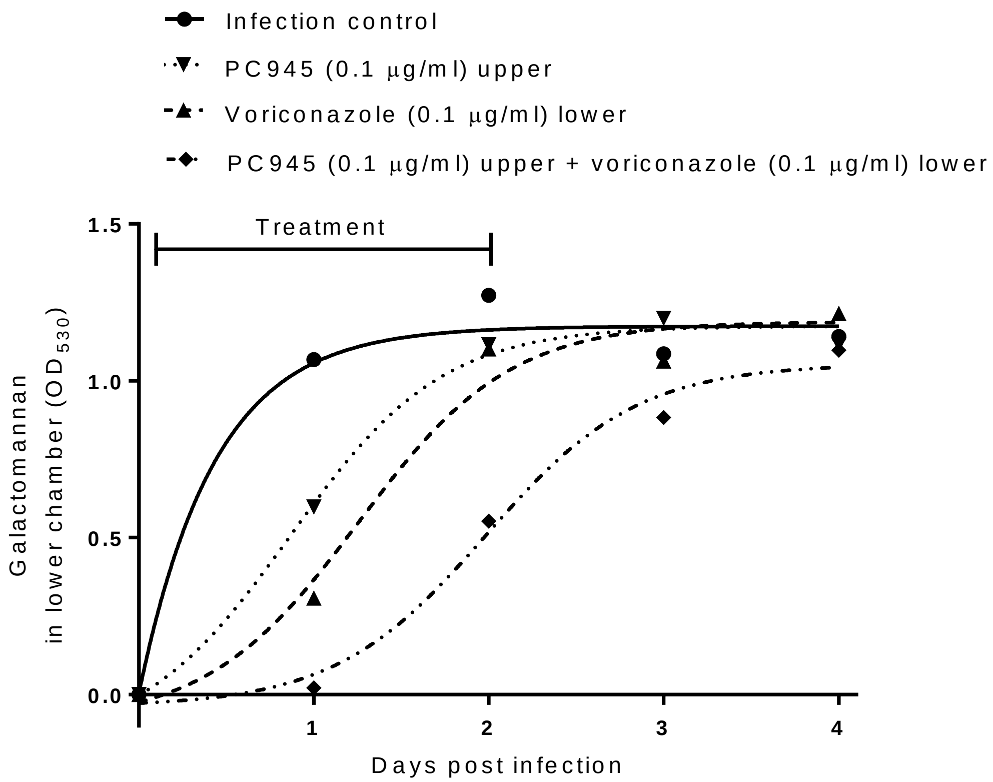

Combined PC945 and voriconazole treatment within an *in vitro* model of the human alveolus. Time course comparison of compounds alone or in combination against azole-sensitive *A. fumigatus* (NCPF2010) penetration of the bilayer. (average of 2 inserts)

Figure S6

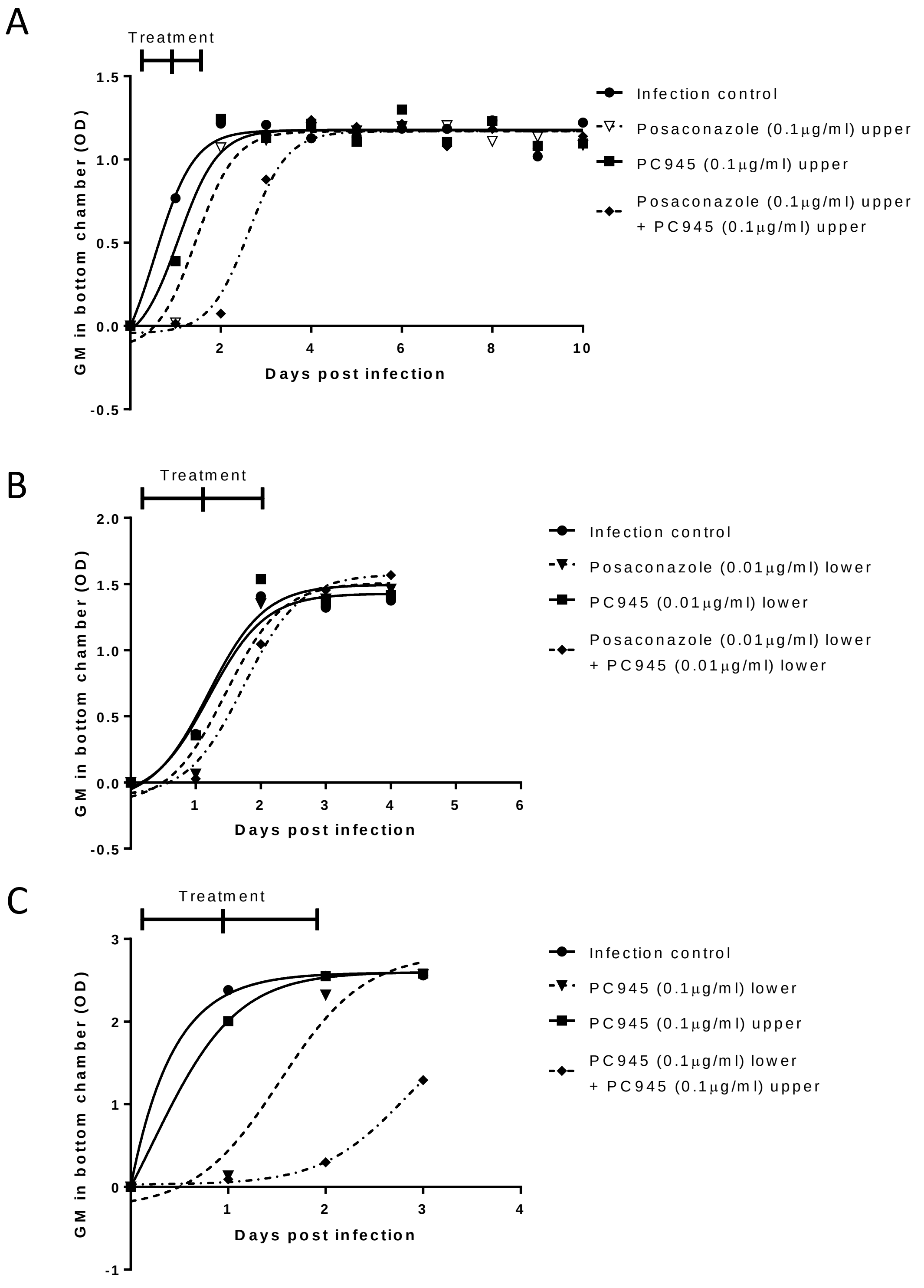

Time course comparison of compounds alone or in combination against azole-susceptible *A. fumigatus (NCPF2010)* penetration of the bilayer.
Both PC945 and posacoanzole were treated in upper chamber only (A) or both PC945 and posacoanzole were treated in lower chamber only (B). PC945 was treated in upper chamber and lower chamber in combination (C). (average of 2 or 3 inserts)

Figure S7

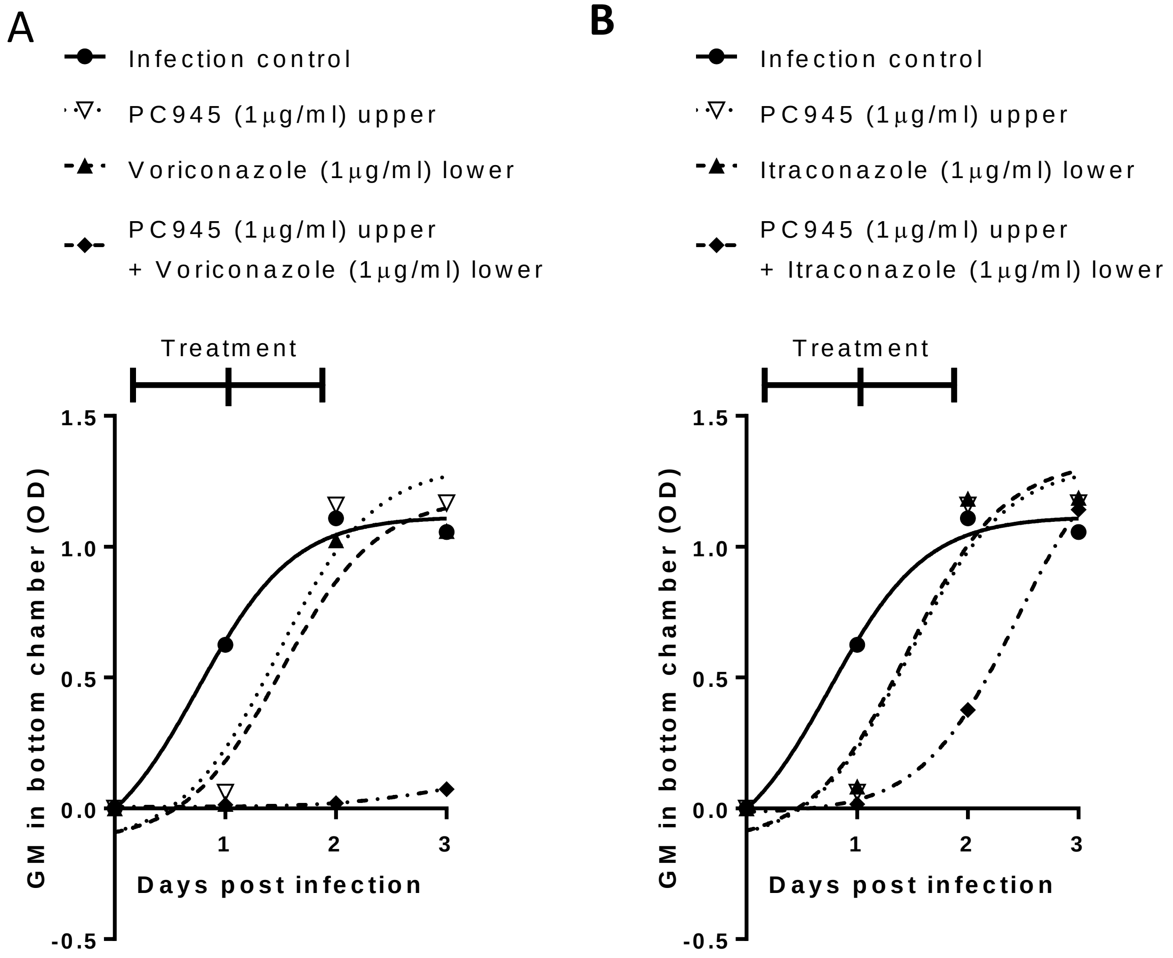

Combined PC945 and voriconazole (A) or itraconazole (B) treatment within an *in vitro* model of the human alveolus. Time course comparison of compounds alone or in combination against azole-resistant TR34/L98H *A. fumigatus* penetration of the bilayer. (average of 2 inserts)
