# Supplementary material for: Antifungal synergy of a topical triazole, PC945, with a systemic triazole against respiratory Aspergillus fumigatus infection
Source: Sci Rep. 2019 Jul 1;9:9482. doi: 10.1038/s41598-019-45890-w (PMC6603190; doi:10.1038/s41598-019-45890-w)
Supplement: Supplementary file 2 — Supplement Tables [file 41598_2019_45890_MOESM2_ESM.pdf]

Supplementary tables

Title

Antifungal synergy of a topical triazole, PC945, with a systemic triazole against respiratory *Aspergillus fumigatus* infection

Thomas Colley[1], Gurpreet Sehra[1], Leah Daly[1], Genki Kimura[2], Takahiro Nakaoki[2], Yuki Nishimoto[2], Yasuo Kizawa[2], Pete Strong[1], Garth Rapeport[1], Kazuhiro Ito[1*]

[1] Pulmocide Ltd, London, SW7 2PG, UK

[2] Laboratory of Physiology and Anatomy, Nihon University School of Pharmacy, Funabashi, Chiba 274-8555, Japan

* Corresponding author: Dr. Kazuhiro Ito; kaz@pulmocide.com.

SUPPLEMENTARY TABLE S1

Susceptibility of PC945 and known antifungals to *A. fumigatus* isolates determined by standard broth microdilution (EUCAST)

	(MIC: µg/mL)				
A.fumigatus strain	PC945	Posaconazole	Voriconazole	Itraconazole	Amphotericin B
NCPF2010	0.063	0.125	1	0.25	2
TR34/L98H	>4	1	>4	>4	0.5
TR_{46}/Y121F/T289A	>16	0.5	>16	>16	0.25

Plates were incubated for 48 h at 35°C, and MIC was determined visually using an azole endpoint.

SUPPLEMENTARY TABLE S2

Quantitative analysis of Susceptibility of PC945 and known antifungals to *A. fumigatus* isolates determined by standard broth microdilution

(µg/mL)	PC945		Posaconazole		Voriconazole		Itraconazole		Amphotericin B	
A.fumigatus strain	IC_{50}	IC_{90}	IC_{50}	IC_{90}	IC_{50}	IC_{90}	IC_{50}	IC_{90}	IC_{50}	IC_{90}
NCPF2010	0.0084	0.010	0.0086	0.014	0.16	0.20	0.016	0.033	0.23	0.48
TR34/L98H	0.034	>1	0.086	0.13	>1	>1	0.22	>1	0.14	0.29
TR_{46}/Y121F/T289A	0.67	>1	0.12	0.72	>1	>1	0.27	0.77	0.088	0.38

Plates were incubated for 48 h at 35°C after which turbidity was assessed by measuring optical density (OD) at 530 nm using a spectrophotometer, and the IC_{50} and IC_{90} values were calculated from the concentration-response curve generated for each test compound.

SUPPLEMENTARY TABLE S3

Antifungal activities of PC945, posaconazole and voriconazole 24h post *A.fumigatus* (NCPF2010 or TR34/L98H) inoculation in bilayer alveolus model

IC_{50}/IC_{90} (µg/mL)	Treatment in upper chamber				Treatment in lower chamber			
	PC945	posaconazole	voriconazole	itraconazole	PC945	posaconazole	voriconazole	itraconazole
NCPF2010	0.17/0.41	0.11/0.15	0.61/0.91	0.074/0.40	NA	0.01/0.036	0.12/0.37	0.09/0.27
TR34/L98H	0.41/1.45	0.16/0.40	1.4/>3	0.37/2.3	NA	<0.01/0.012	0.18/1.1	0.15/0.89

NA: not applicable (not relevant as it is expected only limited systemic exposure), ND: not done

SUPPLEMENTARY TABLE S4

Checkerboard analysis in broth microdilution assay in a microtiter plate for a combination of PC945 and posaconazole (top), PC945 and itraconazole (middle) and PC945 and voriconazole (bottom)

% inhibition		Posaconazole (μg/ml)			
		0	0.008	0.016	0.031
**PC945 (μg/ml)**	0	**0.0**	**9.5**	**14.1**	**62.4**
	0.008	**0.0**	**14.1**	**0.0**	**0.0**
	0.016	**67.2**	**35.0**	**24.9**	**23.0**
	0.031	**88.1**	**39.3**	**90.8**	**85.1**
	0.063	**79.7**	**89.2**	**86.8**	**74.3**

IR value		Posaconazole (μg/ml)			
		0	0.008	0.016	0.031
**PC945 (μg/ml)**	0				
	0.008		**1.49**	**NC**	**NC**
	0.016		**0.50**	**0.35**	**0.26**
	0.031		**0.44**	**1.01**	**0.89**
	0.063		**1.09**	**1.05**	**0.80**

% inhibition		Itraconazole (μg/ml)			
		0	0.031	0.063	0.125
**PC945 (μg/ml)**	0	**0.0**	**0.0**	**67.8**	**74.4**
	0.008	**0.0**	**0.0**	**46.1**	**97.9**
	0.016	**16.9**	**0.0**	**61.8**	**97.1**
	0.031	**56.3**	**96.9**	**97.5**	**98.6**
	0.063	**89.5**	**96.3**	**97.6**	**98.0**

IR value		Itraconazole (μg/ml)			
		0	0.031	0.063	0.125
**PC945 (μg/ml)**	0				
	0.008		**NC**	**0.68**	**1.32**
	0.016		**NC**	**0.84**	**1.23**
	0.031		**1.72**	**1.14**	**1.11**
	0.063		**1.08**	**1.01**	**1.01**

% inhibition		Voriconazole (μg/ml)				
		0	0.063	0.125	0.25	0.5
**PC945 (μg/ml)**	0	**0.0**	**0.0**	**35.0**	**59.9**	**89.7**
	0.008	**0.0**	**0.0**	**0.0**	**72.7**	**99.1**
	0.016	**0.0**	**0.0**	**92.2**	**96.9**	**97.8**
	0.031	**68.9**	**58.3**	**84.8**	**94.8**	**98.4**
	0.063	**89.2**	**97.2**	**95.0**	**98.3**	**97.7**

IR value		Voriconazole (μg/ml)				
		0	0.063	0.125	0.25	0.5
**PC945 (μg/ml)**	0					
	0.008		**NC**	**NC**	**1.21**	**1.10**
	0.016		**NC**	**2.64**	**1.62**	**1.09**
	0.031		**0.85**	**1.06**	**1.08**	**1.02**
	0.063		**1.09**	**1.02**	**1.03**	**0.99**

Inhibition % of fungal growth was calculated using OD values (OD530nm), and IR value (synergy index) was also calculated.
NC: not calculable due to 0% inhibition for both compounds or in combination.

SUPPLEMENTARY TABLE S5 Effects of combination treatment on inhibition of azole-resistant TR_{34}/L98H *A. fumigatus* invasion.

Compartment Treated			Days (% Inhibition)					
Upper	Lower		1	2	3	4	5	6
Posaconazole (1 µg/mL)	DMSO		99.3±0.34[4]	29.5±14.1	1.54±1.44	0.1±0.00	0.65±0.49	1.30±0.78
DMSO	Posaconazole (1 µg/mL)		100±0.43[4]	100±0.40[4]	100±0.40[4]	99.9±0.52[4]	81.4±13.9[4]	0.1±0.00
Posaconazole (1 µg/mL)	Posaconazole (1 µg/mL)		100±0.43[4]	100±0.42[4]	100±0.48[4]	100±0.45[4]	98.9±1.82[4]	0.1±0.00
		SR[1]	1.00±0.00	1.00±0.00	1.00±0.00	1.00±0.00	1.30±0.24	0.21±0.15
Voriconazole (3 µg/mL)	DMSO		30.6±9.46	0.34±0.24	1.48±1.11	0.1±0.00	1.62±0.88	0.90±0.80
DMSO	Voriconazole (3 µg/mL)		100±0.40[4]	100±0.44[4]	99.6±0.18[4]	11.1±3.07	0.62±0.26	1.06±0.96
Voriconazole (3 µg/mL)	Voriconazole (3 µg/mL)		100±0.44[4]	100±0.43[4]	100±0.47[4]	19.5±1.22	15.1±4.38	2.61±1.26
		SR[1]	1.00±0.00	1.00±0.00	1.00±0.00	2.18±0.81	19.8±13.6	12.9±6.47

1:Synergic Ratio (>1: Synergy), 2:p<0.05, 3:p<0.01, 4:p<0.001 vs. infection control

SUPPLEMENTARY TABLE S6 Survival study in *A.fumigatus* (NCPF2010) infected neutropenic immunocompromised mice

S6-1 1st in vivo study (Fig. 3)

		Study survivors On Day 7	Median survival	Log-rank (Mantel Cox) test (p value) (vs infection)	(combination vs.single)
Infection control		0/6 (0%)	5 days		
Posaconazole	1.0 mg/mL po	0/6 (0%)	6.5 days	0.0156	
Posaconazole	10 mg/mL po	4/6 (67%)	undefined	0.0026	
PC945	0.4 mg/mL in	0/6 (0%)	6 days	0.093 (NS)	
PC945 + posaconazole	0.4 mg/mL in + 1.0 mg/mL po	5/6 (83%)	undefined	0.0005	0.0036 vs, POS-1 alone 0.0012 vs PC945 alone

S6-2 2nd in vivo study

		Study survivors On Day 7	Median survival	Log-rank (Mantel Cox) test (p value) (vs infection)	(combination vs.single)
Infection control		0/12 (0%)	5 days		
Posaconazole	1.0 mg/mL po	2/6 (33%)	7 days	0.0087	
Posaconazole	10 mg/mL po	4/6 (67%)	Undefined	0.0011	
PC945	0.4 mg/mL in	1/6 (17%)	6.5 days	0.017	
PC945 + posaconazole	0.4 mg/mL in + 1.0 mg/mL po	5/6 (83%)	Undefined	0.0002	0.022 vs POS-1 alone 0.018 vs PC945 alone

in: intranasally, PO: orally

SUPPLEMENTARY TABLE S7

Effects of repeated prophylactic treatment of PC945 and posaconazole on *A. fumigatus* (NCPF2010) penetration to the human alveolus.

(DFB_{50}: Days)	Posaconazole			PC945		
pre-treatment	-24h	-96 to -24h	-168 to -24h	-24h	-96 to -24h	-168 to -24
0.3µg/mL	1.2	1.9	1.3	<1	2.0	3.0
1µg/mL	2.4	2.6	3.1	2.5	6.8	6.4

Compounds were treated 24h before infection, 96, 72, 48, 24h repeatedly before infection or 168, 144, 120, 96, 72, 48, 24h repeatedly before infection. DFB50 (Days until fungal burden reaches 50% of control) was calculated in each treatment arm.
